# The Integration of Genome-Wide Association Study and Homology Analysis to Explore the Genomic Regions and Candidate Genes for Panicle-Related Traits in Foxtail Millet

**DOI:** 10.3390/ijms232314735

**Published:** 2022-11-25

**Authors:** Xiaodong Liu, Yang Yang, Siyou Hou, Yihan Men, Yuanhuai Han

**Affiliations:** Shanxi Key Laboratory of Minor Crop Germplasm Innovation and Molecular Breeding, College of Agriculture, Shanxi Agricultural University, Taiyuan 030031, China

**Keywords:** foxtail millet, panicle-related traits, genome-wide association study, homology analysis, haplotype analysis

## Abstract

Panicle traits are important factors affecting yield, and their improvement has long been a critical goal in foxtail millet breeding. In order to understand the genetic basis of panicle formation, a large-scale genome-wide association study (GWAS) was performed in this study for six panicle-related traits based on 706,646 high-polymorphism SNP loci in 407 accessions. As a result, 87 quantitative trait loci (QTL) regions with a physical distance of less than 100 kb were detected to be associated with these traits in three environments. Among them, 27 core regions were stably detected in at least two environments. Based on rice–foxtail millet homologous comparison, expression, and haplotype analysis, 27 high-confidence candidate genes in the QTL regions, such as *Si3g11200* (*OsDER1*), *Si1g27910* (*OsMADS6*), *Si7g27560* (*GS5*), etc., affected panicle-related traits by involving multiple plant growth regulator pathways, a photoperiod response, as well as panicle and grain development. Most of these genes showed multiple effects on different panicle-related traits, such as *Si3g11200* affecting all six traits. In summary, this study clarified a strategy based on the integration of GWAS, a homologous comparison, and haplotype analysis to discover the genomic regions and candidate genes for important traits in foxtail millet. The detected QTL regions and candidate genes could be further used for gene clone and marker-assisted selection in foxtail millet breeding.

## 1. Introduction

Foxtail millet (*Setaria italica* L.) is one of the oldest cereal crops and has been domesticated from green foxtail (*Setaria viridis* L.) about 11,000 years ago in northern China [1]. Throughout the history of human farming civilization, foxtail millet has long been cultivated as a staple food in arid and semi-arid regions, particularly in China and India [2]. As a traditional C_4_ crop, foxtail millet has the characteristics of high water use efficiency, drought resistance, and wide adaptability, which makes it a reserve crop for future extreme climate conditions. Millet, the dehusked grain of foxtail millet, is rich in essential amino acids, flavonoids, and a variety of minerals, which have important dietary therapeutic effects in improving human immunity [3]. After more than 10,000 years of planting and cultivation, due to different growth regions, climate, water, soil, and other environmental factors, the foxtail millet germplasm displays immense diversity with rich genetic diversity [4]. In addition, the rapid development of foxtail millet as a C_4_ model crop of Gramineae and the further exploitation of foxtail millet genetic resources and cutting-edge molecular breeding are inevitable due to rising consumer demand for healthy food [5].

In foxtail millet breeding, the improvements in yield and yield-related traits have long been a critical goal, and a key breeding approach for boosting grain output per unit growing area is producing varieties with huge panicles, long branches, high grain numbers, and enlarged grains. Panicle is the important source of foxtail millet yield, and its related traits, such as panicle length (PL), main panicle diameter (MPD), panicle weight per panicle (PWP), grain weight per panicle (GWP), and thousand-grain weight (TGW), directly affect the yield formation of foxtail millet. Therefore, improving panicle-related traits is always an important research area in foxtail millet breeding, especially in yield breeding. In addition to being directly related to yield, panicle-related traits, such as panicle length, affect the lodging resistance of foxtail millet. Although the foxtail millet industry in China has made great progress after 40 years of scientific breeding, the genetic basis for the formation of important agronomic traits in millet, especially the panicle architecture, is not clear.

Gramineae panicle architecture is influenced by a number of factors, including plant growth regulators, photoperiod, flower organs, grain development, etc. The most direct determinant is the fate of meristems and the transformation of branching meristems into spikelet meristems [6]. As a classical pathway for maintaining the growth and differentiation of plant inflorescence meristems, a large number of functional genes in the CLAVATA-WUSCHEL feedback loop pathway have been cloned and functionally analyzed in Rice, such as *FON1*, *FON4* and *OsWUS* [7,8,9], and many MADS-box gene family members, including *OsMADS14*, *OsMADS15*, *OsMADS18*, and *OsMADS34(PAP2)*, were shown to affect panicle architecture by regulating the meristem size and specification of meristem identities [10,11]. Unlike the CLAVATA-WUSCHEL pathway, which directly regulates the growth and differentiation of meristems, plant growth regulators such as cytokinin, auxin, and gibberellin affect the panicle structure, mainly by regulating the identity, initiation, and enlargement of inflorescence meristems. A loss of function in the *OSCKX2* gene, which encodes the cytokinin oxidase dehydrogenase, causes cytokinin to accumulate in the inflorescence meristems, resulting in more branching and spikelets [12]. In contrast, the *OSH1* gene in rice regulates the relative content of GA and cytokinin to promote meristem identities [13].

The majority of gene-based panicle architecture research studies have been conducted on rice and maize, with only a few examining foxtail millet. Doust et al. (2005) detected 14 QTLs associated with a primary branch number, primary branch density, spikelet number, and bristle length by QTL analysis in a bi-parental population derived from foxtail millet and green foxtail [14]. In other two QTL mapping studies, 12 and 32 QTL were also identified with some panicle-related traits, such as PWP, GWP, TGW, etc. [15,16]. Most of these studies were based on a small number of polymorphic loci, and fewer QTL were identified. A recent QTL mapping study based on 12 environments identified 159 QTLs associated with panicle-related traits, which improved our understanding of the genetic basis for panicle and yield formation in foxtail millet [17]. However, QTL results based on bi-parental populations are heavily dependent on the genetic background of the population and environment, which greatly limits the wide adaptability and stability of these QTLs in foxtail millet breeding programs [18].

While these preliminary studies have strengthened our understanding of the genetic basis for foxtail millet panicle-related traits, it is not necessary to use these observations to mine important genes regulating panicle-related traits and to improve yield in foxtail millet. Recently, with the rapid developments in DNA sequencing technology, a genome-wide association study (GWAS) has become a powerful tool for analyzing the genetic basis of important traits, especially for complex traits controlled by multiple genes. It has been widely used in QTL and in the genetic discovery of different traits in many crops, and achieved good results. The genetic basis of some complex traits, such as leaf and stripe rust resistance in break wheat, morphological traits exposed to drought in barley, agronomic traits in rice, and major biofuel traits in sorghum, has been analyzed by GWASs to some extent [19,20,21,22]. There are relatively few GWASs on foxtail millet, and only three large-scale GWASs have been reported to mine QTL and genes related to agronomic traits and metabolites in foxtail millet [5,23,24]. In addition, a large number of studies have confirmed the existence of convergent selection in the domestication and modern breeding of gramineous crops, and a series of genes, including *TaKAO-4A*, have been found in wheat by large-scale homology analysis, which are key candidate genes for the rice-like selection and regulation of agronomic traits [18]. Therefore, the homology analysis of genes regulating important agronomic traits in model species such as rice is also a way to find important functional genes in other species, especially for minor crops such as foxtail millet.

In this study, to understand the genetic basis of panicle formation in foxtail millet, a large-scale GWAS of 407 accessions was constructed using six panicle-related traits and genome resequencing data. The objectives of this study were to (1) evaluate the variation of six panicle-related traits under different environments and analyze their correlation; (2) genotype 407 foxtail millet accessions using genome resequencing data; (3) mine important genome regions affecting the panicle-related traits of foxtail millet based on GWAS; and (4) mine key candidate genes for panicle-related traits in foxtail millet based on homology alignment and haplotype analysis. This study will provide comprehensive and accurate information which will improve our understanding of the genetic basis of foxtail millet spike formation, and will help to mine important functional genes of millet.

## 2. Results

### 2.1. Phenotypic Characteristics of Six Panicle-Related Traits in Foxtail Millet

**Figure 1:**
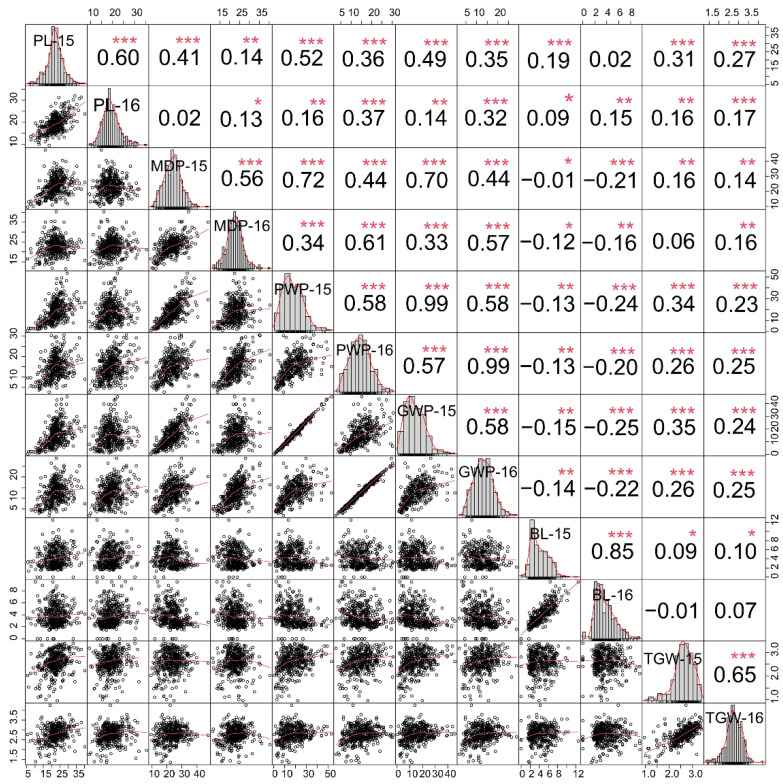
Phenotypic characteristics of six panicle-related traits of 407 foxtail millet accessions in two environments (2015 and 2016). The scatter plots and the correlation coefficients of six panicle-related traits are listed below and above the diagonal, respectively. PL, MPD, PWP, GWP, BL, and TGW represent the panicle length, main panicle diameter, panicle weight per panicle, grain weight per panicle, bristle length, and thousand-grain weight, respectively. *, **, and *** represent significant correlations at *p* < 0.05, *p* < 0.01, and *p* < 0.001 levels, respectively.

A total of 407 foxtail millet accessions from different original producing areas were investigated for spike-related traits for two consecutive years (Table S1). Six panicle-related traits all showed approximate normal distributions under two summer cropping seasons in 2015 and 2016 (Figure 1). Of which, the panicle length (PL), main panicle diameter (MPD), panicle weight per panicle (PWP), grain weight per panicle (GWP), and thousand-grain weight (TGW) were more consistent, while the bristle length (BL) showed a weak skewed distribution, which confirmed that these traits are all complex traits controlled by multiple genes. The six panicle-related traits had high broad-sense heritability (all above 0.7) in two environments, with a minimum of PWP (0.73) and a maximum of BL (0.91), which indicated that these traits are mainly affected by genotypes (Table 1). The coefficient of variation (CV) of different traits showed great differences among different genotypes. Among them, the CVs of TGW (19% and 16%) and PL (20% and 20%) were low, while the CVs of the remaining four traits were high, and the CV of GWP in 2015 reached 57%. In general, these accessions and their six panicle-related traits all showed significant differences in genotype and environment, indicating that the natural population of foxtail millet constructed with these 407 accessions was suitable for an genome-wide association study of these six panicle-related traits.

To clarify the relationships between these panicle-related traits, the Spearman approach was used to examine the basic correlations of six panicle-related traits in two environments (Figure 1). Other panicle-related traits, such as the PL, MPD, PWP, GWP, and TGW, were all significantly positively correlated, with the exception of the BL, which exhibited a weak correlation with other traits. In particular, the correlation coefficient between the PWP and the GWP was more than 0.95 under the same environment (*p* < 0.001), and the correlation coefficient between the MDP, the PWP, and the GWP was more than 0.7 (*p* < 0.001). The high correlation coefficients of these traits suggested that they might be regulated by the same multiple quantitative trait loci (QTL) or genes.

### 2.2. Genotyping, Population Structure, Principal Component, and Linkage Disequilibrium Analysis

**Figure 2:**
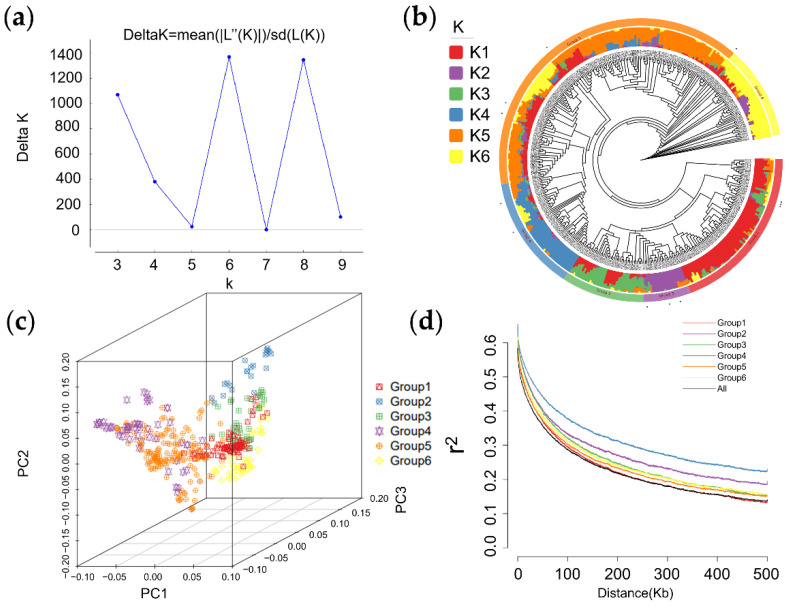
Genotypic characteristics of 407 foxtail millet accessions based on SNP variation information. (**a**) A line chart of Δk values performed by Structure Harvester software; (**b**,**c**) a phylogenetic tree and principal component analysis of 407 foxtail millet accessions, respectively; (**d**) the attenuation distance of the linkage disequilibrium of foxtail millet.

**Figure 3:**
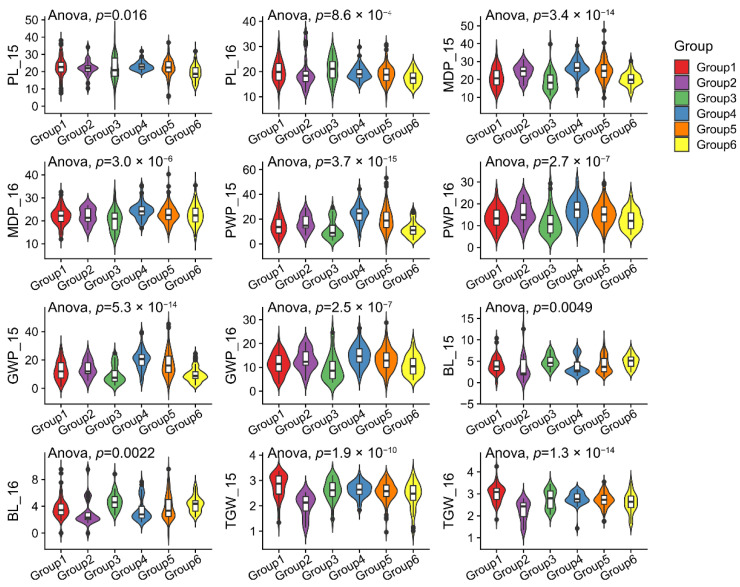
The phenotypic distribution of six panicle-related traits of foxtail millet accessions from different sub-groups. These sub-groups were obtained by population structure and phylogenetic tree analysis based on genomic SNP variation information. The violin plots were drawn using the R backage ggplot2, and the width of the violin represents the distribution density of the phenotypic values.

After filtering out the SNP loci with a minor allele frequency (MAF) of <0.05 and missing data >20%, a total of 706,646 high-polymorphism SNP loci were obtained to genotype 407 foxtail millet accessions. The population structure was analyzed by FastStructure and k = 6 was chosen as the best fraction of the population structure after the Δk value was analyzed by Structure Harvester software (Figure 2a). Phylogenetic analysis showed that the 407 foxtail millet accessions were classified six 6 subgroups (Groups 1–6), which included 80, 22, 39, 54, 161, and 44 accessions, respectively. Twelve wild resources were distributed in Groups 1, 2, 4, and 5, with the exception of Groups 3 and 6 which were rich in modern cultivars. (Figure 2b). Similarly, six subgroups could be well classified by principal component analysis (Figure 2c). The linkage disequilibrium (LD) analysis showed that the LD attenuation distance of foxtail millet materials in different subgroups was inconsistent (Figure 2d). The LD of accessions in Group 4 attenuated slowly, while the LD of accessions in Group 1 attenuated most quickly. Genomic regions within 100 kb were regarded as a QTL region based on prior studies and the LD attenuation of all accessions [5,24]. Additionally, foxtail millet accessions of different subgroups also showed significant differences in six panicle-related traits (Figure 3). For example, accessions of Group 3 showed lower PWPs and GWPs in two environments, while those of Group 4 showed higher PWPs and GWPs. There is an interesting polarity between these two classes of materials with regards to the BL, suggesting that the domestication processes of some panicle-related features may have simultaneously occurred. These results indicate that there were significant differences among the six subgroups with phenotypic traits and genetic background.

### 2.3. Quantitative Trait Nucleotides (QTNs) and QTL Regions for Six Panicle-Related Traits

**Figure 4:**
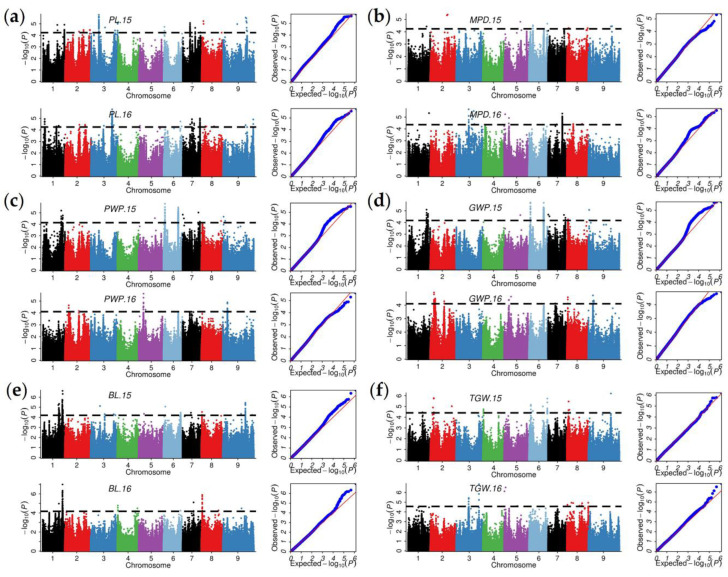
Manhattan and quantile–quantile plots performed by Tassel 5.0 to identify quantitative trait nucleotides (QTNs) significantly associated with six panicle-related traits in two environments. (**a**–**f**) The panicle length (PL), main panicle diameter (MPD), panicle weight per panicle (PWP), grain weight per panicle (GWP), bristle length (BL), and thousand-grain weight (TGW), respectively.

The results of GWAS using the MLM model of Q (population structure) + K (kinship) strategy showed that the MLM model had good adaptability, and did not show obvious insufficient correction (Figure 4). The best linear unbiased prediction (BLUP) is one of statistical methods used by linear mixed models to predict random effects. The BLUP of random effects is similar to the best linear unbiased estimates (BLUEs) of fixed effects. The BLUP method can integrate multiple environmental data to remove environmental effects and obtain stable genetic phenotypes of individuals; therefore, it is often used as a set of phenotypic values for multi-environment QTL mapping and GWAS analysis. A total of 1830 QTNs were detected to be significantly associated with six panicle-related traits in multiple environments (2015, 2016, and BLUP) (Table 2). The number of QTNs related to different traits varied under different environments, explaining 0.01–26.78% of phenotypic variation. Among them, more QTNs were detected with BLUP values as phenotypic data than in the two natural environments, which was related to the fact that BLUP integrated the phenotypic values of two natural environments. In terms of two natural environments, the maximum number of QTNs significantly related to fact that the GWP in 2015 was 270, while the minimum number of QTNs significantly related to the BL in 2016 was 24. Large differences in the number of these QTNs were associated with phenotypic data for these traits, with the GWP likely being controlled by more genes and the BL likely being affected by relatively few genes. Phenotypic evidence revealed that the GWP showed a standard normal distribution in both environments, while the BL showed a weak partial distribution.

**Figure 5:**
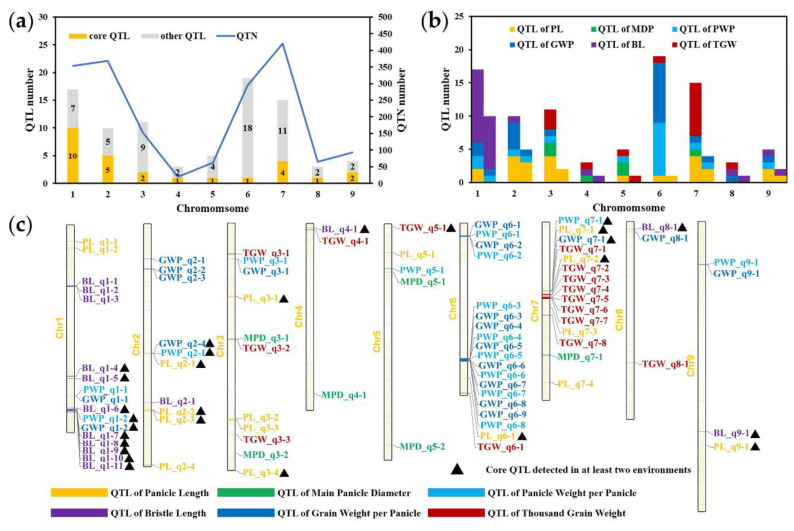
QTL regions significantly associated with six panicle-related traits. (**a**) The number of QTN and QTL regions associated with six panicle-related traits; (**b**) the number of QTL regions associated with each panicle-related trait; (**c**) the distribution of QTL regions on chromosomes. The columns on the right represent the number of core QTL regions in (**b**), and the core QTL regions in (3) are marked with black triangles.

Based on the LD decay distance in this study and previous studies, genomic regions within 100 kb that contained at least four significant QTNs were considered to be QTL regions [5,24]. After detailed artificial screening, 87 QTL regions were composed of these significantly associated QTNs (Figure 5a,b). These QTL regions were named according to their location on the chromosome and their associated traits. For example, PL_q1-1 represented the first QTL region on the chromosome 1 that was significantly associated with the PL, while MPD_q5-2 represented the second QTL region on chromosome 5 that was significantly associated with the MPD (Figure 5c). Of these 87 QTL regions, 17, 6, 15, 19, 15 and 15 QTL regions were significantly associated with the PL, MPD, PWP, GWP, BL, and TGW, respectively (Figure 5c, Table S2). On average, these QTL regions explained 8.03% of the phenotypic variation, with TGW_q6-1 explaining only 0.36% of the TGW variation and PWP_q1-2 explaining 25.45% of the PWP variation. The distribution of these QTL regions on chromosomes was uneven, with 17, 19, and 15 regions identified on chromosomes 1, 6, and 7, respectively, while only 3 QTL regions were identified on chromosome 4. These results suggest that these three chromosomes may contribute more to panicle formation in foxtail millet. Moreover, the number of QTNs in different QTL regions ranged from 4 to 190, which confirmed that the current QTL regions contained hot spots with high confidence that affected the panicle-related traits in foxtail millet.

To identify these high-confidence hot spots, the QTL regions detected in at least two environments were considered as stable core QTL. A total of 26 core QTL regions were obtained, of which, 9, 3, 3, 11, and 1, were related to the PL, PWP, GWP, BL, and TGW respectively (Table 3). Similar to the distribution of QTL regions on chromosomes, the number of core QTL on chromosomes 6 and 7 was 18 and 11, respectively. Moreover, most of the QTL regions on chromosome 6 were related to the PWP and the GWP, with most of the QTLs on chromosome 7 being significantly associated with the TGW (Figure 4b,c). These core QTL regions explained a minimum of 4.55% of the phenotypic variation and an average of 12.74% of the phenotypic variation. In addition, a large number of QTL regions significantly associated with different traits, such as PWP_q1-2, BL_q1-6, GWP_q2-4, and PWP_q2-1, were co-located on the genome, which was consistent with the extremely significant correlations among these traits. Moreover, it was confirmed that these QTL regions may affect several ear traits at the same time (Figure 4c). Although other QTL regions were temporarily excluded due to their low stability between environments, they were considered secondary candidate loci for subsequent analysis. To verify the accuracy of the QTL regions identified in this study, we compared QTL regions detected in this study with the reliable QTLs in previous GWAS and QTL mapping studies based on their physical locations (Table 4). Thirty-nine QTL regions were overlapped with, or close to, previously reported QTLs associated with panicle-related traits. For example, PWP_q6-1, GWP_q6-1, PWP_q6-2, and GWP_q6-2 (2.535–2.794 Mb) were overlapped with qPWP6.2 (2.631–3.434 Mb), PWP_q7-1, and GWP_q7-1 (13.851–13.961 Mb), but were closed to qGWP7.1 and qPWP7.2 (13.405–13.643 Mb).

### 2.4. Candidate Gene Mining and Haplotype Analysis Based on Homology Comparison Strategy

**Figure 6:**
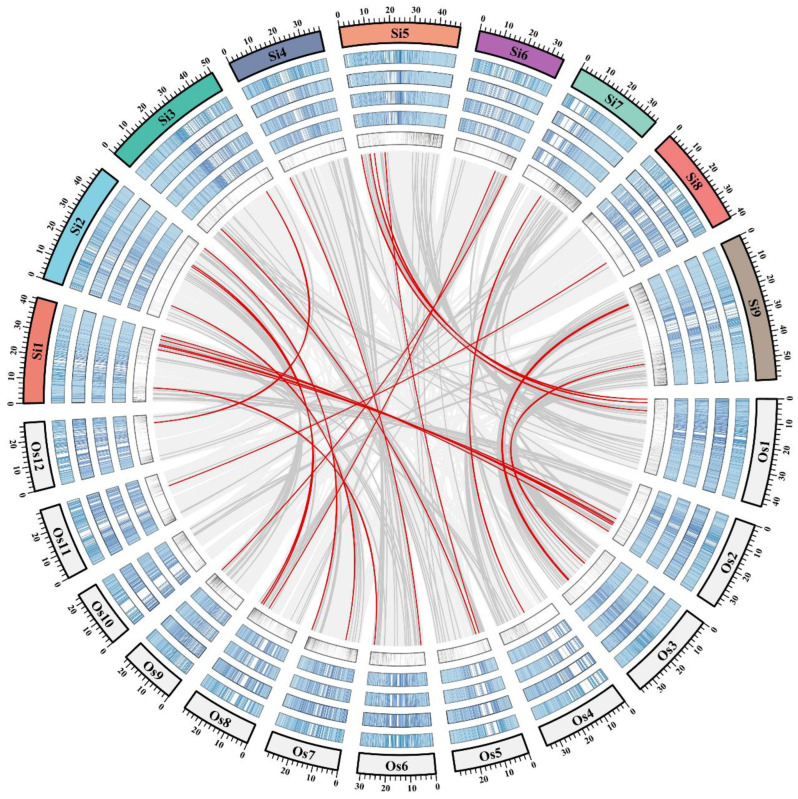
Collinearity analysis of orthologous genes related to yield- and panicle-related traits in foxtail millet and rice. The outermost circle represents the chromosome combination of millet (Si) and rice (Os), and is marked with different color backgrounds. The next four circles represent the gene expression levels in the roots, stems, leaves, and panicles of foxtail millet and rice, respectively. All expression data were derived from the multi-omics database for *Setaria italica* (MDSi, http://foxtail-millet.biocloud.net/home (accessed on 10 May 2022)) and the rice expression database (RED, http://expression.ic4r.org/index (accessed on 10 May 2022)), and the average TPM values within 100 kb windows were used to display gene expression levels. The innermost circle represents the gene density. The light gray, gray, and red lines represent the collinear regions between millet and rice, the orthologous genes for yield and panicle-related traits, and the 27 candidate orthologous genes in the QTL regions, respectively.

Few studies have been carried out on the genetics of panicle-related traits in foxtail millet, but rice has been used as a model crop for the functional studies of cereal crops, and many genes that control the yield and panicle-related traits have been cloned from rice (Table S3). After a detailed literature investigation, more than 300 important functional genes related to yield-related traits were obtained in rice and 264 orthologs were also identified in foxtail millet, which were located in collinear regions in rice and foxtail millet (Table S4 and Figure 6). Among them, 27 genes were located in the QTL regions, which might affect the panicle-related traits in foxtail millet.

**Figure 7:**
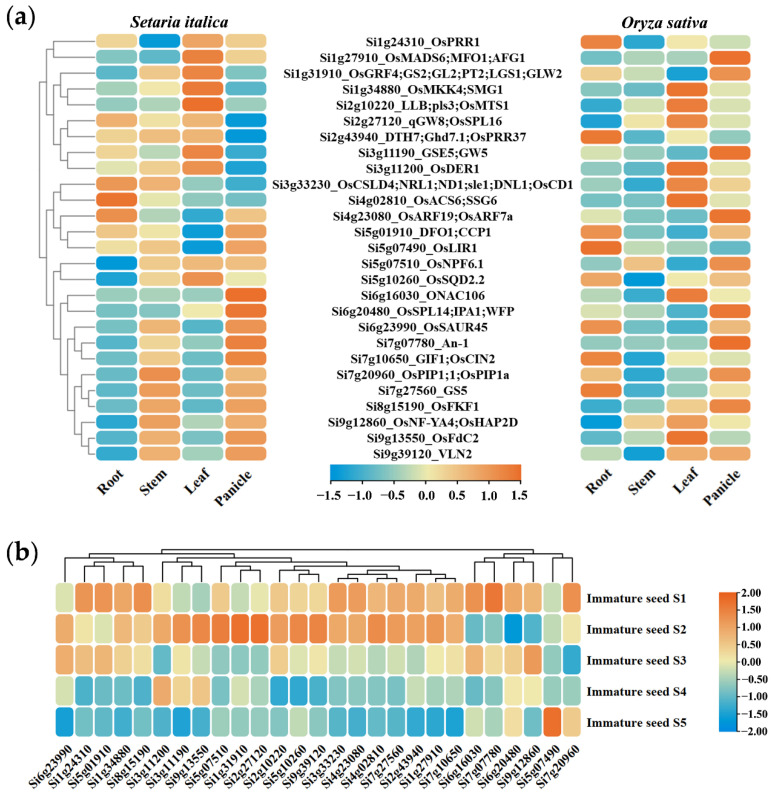
Expression characteristics of 27 pairs of orthologous genes in foxtail millet and rice. (**a**) The expression data of 27 pairs of orthologous genes in the roots, stems, leaves, and panicles of foxtail millet and rice were derived from the multi-omics database for Setaria italica (MDSi, http://foxtail-millet.biocloud.net/home (accessed on 10 May 2022)) and the rice expression database (RED, http://expression.ic4r.org/index (accessed on 10 May 2022)), respectively. (**b**) The expression patterns of 27 candidate genes in foxtail millet during grain development (S1 to S5). All expression data were derived from the multi-omics database for Setaria italica (MDSi, http://foxtail-millet.biocloud.net/home (accessed on 10 May 2022)). From blue to red, the expression level increases from low to high, and Log2 (TPM + 1) values are used to characterize the expression levels.

Based on the public expression data of foxtail millet and rice, the expression patterns of these candidate genes and their rice orthologs in four major tissues were compared. The correlation coefficient on the expression patterns between the 27 candidate genes and their rice orthologs was 0.617 at the *p* < 0.01 level, which indicates that these genes might have conserved functions to some extent. These orthologs showed similar expression patterns in foxtail millet and rice, especially in the panicle (Figure 7a). Further analysis showed that these candidate genes were mainly expressed at a high level in the early grain development (S1 and S2) of foxtail millet, suggesting that these genes may be involved in grain development to regulate the formation of panicle-related traits (Figure 7b).

**Figure 8:**
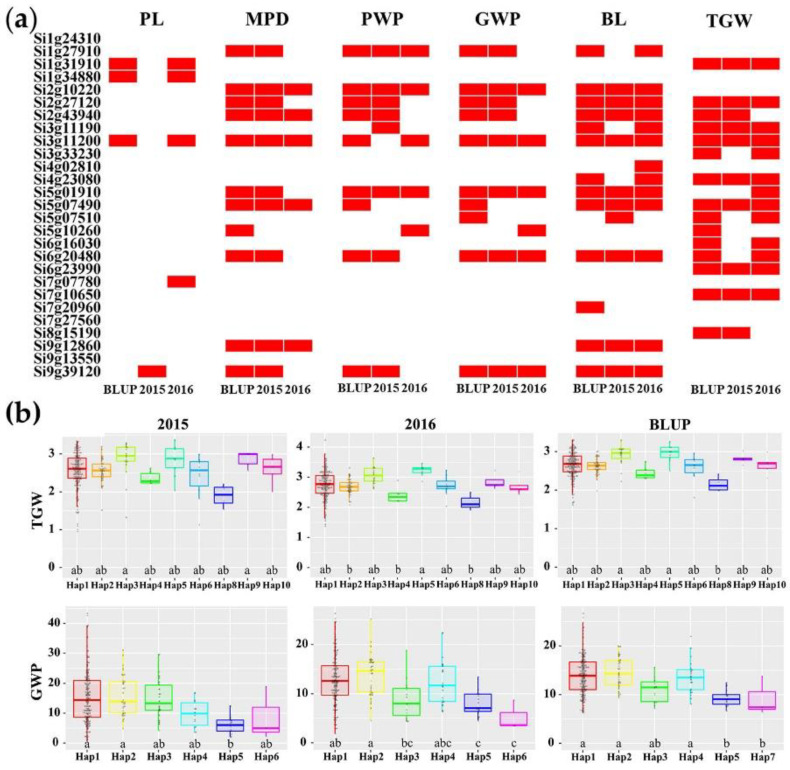
Haplotype analysis of 27 candidate genes in QTL regions in two environments and BLUP. (**a**) The contribution of excellent haplotypes of 27 candidate genes to phenotypes in multiple environments. The red block represents that excellent haplotypes of candidate genes have significant contributions to the phenotype in this environment at *p* < 0.01. (**b**) The effects of different haplotypes of two representative genes *Si5g01910* and *Si3g11190* on the TGW and the GWP. Above and below represent the effects of *Si5g01910* on the TGW and *Si3g11190* on the GWP in two environments (2015, 2016) and BLUP, respectively. PL, MPD, PWP, GWP, BL, and TGW represent the panicle length, main panicle diameter, panicle weight per panicle, grain weight per panicle, bristle length, grain yield, and thousand-grain weight, respectively. Letters (a–c) in (**b**) represent significant differences.

In addition, haplotype analysis showed that, except for three genes, different haplotypes of the other 24 genes affected at least one panicle-related trait in at least two environments (including BLUP) (Figure 8a). Among the 24 genes, 13 genes affected at least two panicle-related traits in at least two environments. Notably, different haplotypes of *Si3g11200* affected all six panicle-related traits in at least two environments, and the MPD, PWP, GWP, BL, and TGW were also affected in at least two environments by *Si2g27120*, *Si2g43940*, and *Si6g20480*. The effects of these candidate genes in foxtail millet were quite similar to their effects in rice. For example, *Si3g11200* was located in the QTL region of the PWP and the GWP, and haplotype analysis showed that it affected all panicle-related traits. Its orthologue *OsDER1* in rice affected grain size and 1000-grain weight by regulating grain development. Although *Si7g10650* was found to effect millet’s 1000-grain weight in all three environments, its rice homologous gene OsCIN2 influenced grain size and yield via controlling endosperm and starch content (Table 5). In addition, the stable contribution of these candidate genes to panicle-related traits in multiple environments could be used as the optimal loci for marker-assisted selection in order to improve the yield in foxtail millet.

## 3. Discussion

Foxtail millet is one of the earliest domesticated cereal crops and is widely cultivated in arid and semi-arid regions such as China and India [2]. Its shell-breaking product, millet, is rich in essential nutrients, such as amino acids, flavonoids, and minerals, and has important dietary therapeutic effects in improving human immunity [3]. However, unlike major crops such as wheat, rice, and maize, foxtail millet is not intensively bred. The development of foxtail millet as a C_4_ photosynthetic model crop and the demand of consumers for healthy food make the deep excavation of foxtail millet genetic resources and modern molecular breeding especially urgent [5].

Panicle, as an important source of foxtail millet yield formation, including panicle weight, panicle length, panicle diameter, thousand-grain weight, and other related traits, directly affects the grain yield of foxtail millet. Here, we evaluated the broad-sense heritability of these six panicle-related traits and found that the heritability rates of the BL (0.91) and PL (0.78) were higher than those of the PWP (0.73) and the GWP (0.74), which was consistent with a previous study of panicle-related traits [17]. Furthermore, six panicle-related traits all showed significant correlations under both environments (Figure 1). Specifically, the PL and the MPD were significantly and positively correlated with the PWP and the GWP, which confirmed the contribution of panicle architecture to yield. Therefore, improving the panicle architecture, especially those traits with high heritability such as the PL and MPD, is an important way to increase the yield of foxtail millet.

Although some genome-wide association studies have reported on the agronomic traits and metabolomes of foxtail millet, there are few studies on the genetic basis of panicle-related traits in foxtail millet based on large-scale variation information [5,24]. In this study, we performed a genome-wide association study on six panicle-related traits of 407 foxtail millet based on genome resequencing data. The phenotypic characteristics of six panicle-related traits in two environments and BLUP were analyzed in detail. These traits were all affected by the environment based on genotype regulation, which was consistent with previous studies on foxtail millet or other crops [17,24]. Several panicle-related traits, including PWP, GWP, and TGW, showed very high correlations in multiple environments, which indicated that these traits might be regulated by some of the same genes. This was also confirmed by the co-localized QTL regions affecting different traits and the co-effects of the different haplotypes of candidate genes on multiple traits.

After filtering out the SNP loci with relatively poor polymorphism and data quality, we still retained more than 700,000 SNP loci (with a density of about 1.5/1 kb) for genotyping and GWASs. LD analysis revealed a linkage disequilibrium attenuation distance of about 100 kb in foxtail millet, which was consistent with two previous GWAS studies [5,24]. Previous studies have confirmed that GWASs based on high-density SNP variation information had a huge advantage in mining major QTL, but for those complex traits controlled by multiple genes, the detection rate and accuracy of those inefficient QTL would be greatly reduced, while the large number of SNPs significantly associated with traits also increased the difficulty of candidate gene mining [20]. To avoid this problem, the significant QTNs located in the linkage blocks were merged to improve the detection rate of QTL regions and facilitate the mining of candidate genes. As a result, 87 QTL regions were found to be associated with six panicle-related traits, and 39 of them were overlapped with, or close to, previous reported QTLs. Although these QTLs were identified by different traits, they could be considered reliable according to the highly significant correlation of panicle-related traits. Overall, co-location with previous QTLs confirmed the reliability of this study, and these new QTLs were also important loci for improving panicle traits in foxtail millet. Among the 87 QTL regions, 27 QTL regions with stable multiple environments were considered core QTL regions. These core QTL regions were mainly concentrated on chromosomes 1, 2, and 7, which was consistent with previous studies, indicating that there were important candidate genes regulating panicle-related traits on these chromosomes [24]. These core QTL regions can be used as important genomic resources for further breeding improvement of panicle- and yield-related traits and functional gene mining in foxtail millet. In addition, our team is comparing polymorphisms within important QTL regions in modern cultivars, and based on the excellent haplotype information obtained in this study, we are developing some convenient and fast KASP markers for other millet natural population validation and molecular marker-assisted selection.

As a minor crop, the research on functional genes in foxtail millet is limited, which makes functional gene mining in foxtail millet more difficult. A recent genome-wide comparative analysis detected 490 homologous gene pairs subjected to convergent selection in rice and maize, confirming the existence of large-scale convergent selection on the rice and maize genomes [54]. Another large-scale meta-QTL analysis in wheat also unearthed the key gene *TAKAO-4A*, which controls grain morphology, by comparing orthologous genes between rice and wheat [18]. Although only a few functional genes have been reported in foxtail millet, several genes cloned in foxtail millet have been reported to have similar functions to their rice orthologs. *SiMADS34*, encoding an E-class MADS-box transcription factor, was proved to influence grain yield in foxtail millet by regulating the inflorescence architecture, which was similar to the function of its homologous gene *OSMADS34* in rice [10,55]. The *SiCCD1* and *OsCCD1* genes encoding carotenoid cleavage dioxygenase 1 affect grain color and carotenoid content by regulating lutein degradation in millet and rice, respectively [56,57]. Furthermore, the orthologs of some well-known rice genes such as *OsSD1*, *OsPSY1*, and *OsAUX1* were also found to exhibit similar expression patterns and functions in foxtail millet [5,58,59,60]. All these indicate that there might be a large number of important functional genes with conserved functions among different crops of Gramineae. Here, more than 300 rice genes related to yield- and panicle-related traits and 200 foxtail millet orthologs were identified in rice and foxtail millet, respectively. They were all located in the collinearity regions between rice and foxtail millet. Of these, twenty-seven foxtail millet genes were located in QTL regions associated with panicle-related traits. Haplotype analysis showed that 24 genes were significantly associated with at least one panicle-related trait in at least two environments and 15 genes were associated with at least one panicle-related trait in all environments. In addition, 10 genes were significantly associated with more than three panicle-related traits in at least two environments, of which one, three, and three genes were associated with six, five, and four traits, respectively. In general, the effect of 89% (42/27) of candidate genes on panicle-related traits of foxtail millet was verified by haplotype analysis, which confirmed the reliability of the strategy of rapidly mining candidate genes based on homology alignment combined with haplotype analysis [18].

These candidate genes regulate panicle- and yield-related traits in rice through multiple pathways including plant growth regulators (GAs, e.g., brassinolide and auxin), photoperiod, flower and panicle development, photosynthesis, etc. For example, *OsGRF4* (*Si1g31910*) [29], *OsARF19* (*Si4g23080*) [38], OsSAUR45 (*Si6g23990*) [45], *GSE5*(*Si3g11190*) [34,60], and *VLN2* (*Si9g39120*) [53] were all involved in the synthesis or transduction of GA, brassinolide, and auxin to regulate flower, panicle, and grain development. In addition, *DTH7* (*Si2g43940*) [33], *OsMADS6* (*Si1g27910*) [28], *OsNF-YA4* (*Si9g12860*) [51], *OsFKF1* (*Si8g15190*) [50], and *DFO1* (*Si5g01910*) [39] affected grain morphology and grain weight by participating in photoperiod or directly regulating flower development and panicle formation in rice. *OsACS6* (*Si4g02810*) [37], *GIF1*(*Si7g10650*) [47], and *OsSQD2.2* (*Si5g10260*) [42] directly regulated grain size by regulating grain filling and sugar metabolism; *OsLIR1* [40] (*Si5g07490*) and *OsFdC2* (*Si9g13550*) [52] indirectly affected panicle- and yield-related traits by affecting photosynthetic capacity. In addition, the 27 genes contained several homologous genes of rice grain morphology were widely reported, such as *qGW8* (*Si2g27120*) [32], *OsDER1* (*Si3g11200*) [35], *GS5* (*Si7g27560*) [49], etc. Although *Si7g07780*, the foxtail millet orthologous gene of rice awning length gene *An-1*, was found in the QTL region of TGW, it did not show significant association with the foxtail millet BL, which indicates that BLs might be controlled by other genes. Overall, we identified multiple high-confidence candidate genes associated with panicle-related traits in foxtail millet by comparison of orthologous genes between rice and foxtail millet and haplotype analysis, thus confirming the reliability of this strategy. The breeding of staple crops such as rice and maize has gradually entered the stage of molecular design breeding or intelligent breeding. Through the construction and coupling of several molecular modules by regulating the yield, plant architecture, and other important traits in rice, a number of breakthrough varieties and hybrids have been developed, which greatly increased rice yield. However, for foxtail millet, the mining of its functional genes and excellent haplotypes could not meet the requirements of molecular design breeding. This study proposed and verified a strategy based on the integration of GWASs, homologous comparison, and haplotype analysis to discover the genomic regions and candidate genes for important traits in foxtail millet. Furthermore, this strategy can be further expanded to accelerate the important functional gene mining and molecular breeding of foxtail millet on the principle basis of the convergent selection of gramineous crops. Both large-scale targeted association analysis based on the minor crop species model orthologs and analysis based on key metabolic pathway genes are effective methods that can be tried. Overall, this study provides an initial demonstration of how the results of model crop studies can be used to facilitate the mining of functional genes in minor crops. These important candidate genes and their excellent haplotypes will be further used in the functional gene mining and molecular breeding of foxtail millet.

## 4. Materials and Methods

### 4.1. Plant Materials

To ensure the broad diversity of foxtail millet materials, a total of 407 genotypes, including landrace, wild species, modern cultivar, and foreign germplasm, were used for GWASs on panicle-related traits (Table S1). All materials were obtained from the Institute of Crop Sciences, the Chinese Academy of Agricultural Sciences, and grown during two summer cropping seasons (May to October of 2015 and 2016) on the experimental farm of Shanxi Agricultural University, Taigu, Shanxi, China (37°25′ N, 112°35′ E). The field experiment was carried out in a randomized complete block design with three replicates. Each material was planted in three rows, each 5 m in length with a 25 cm space between rows. Field management followed the local standard foxtail millet agronomic management practice. Before the compound fertilizer of nitrogen, phosphorus, and potassium was planted, weeds were controlled and fungicide and pesticide were sprayed in a timely fashion.

### 4.2. Measurement and Statistical Analysis of Panicle-Related Traits

After harvest, 10 panicles of each foxtail millet genotype were separated for the measurement of five panicle-related traits, including panicle length (PL, cm), main panicle diameter (MPD, cm), panicle weight per panicle (PWP, g), grain weight per panicle (GWP, g), and bristle length (BL, mm). Thousand-grain weight (TGW, g) was measured after seed threshing and drying. All measurement of these traits followed the Descriptors and Data Standard for Foxtail Millet [*Setaria italica* (L.) *Beauv*.].

The best linear unbiased prediction (BLUP) values of six panicle-related traits in two environments (2015 and 2016) were calculated by R package Lme4, and ANOVA analysis was performed by SAS 8.0 (SAS Institute Inc., Cary, NC, USA) [61]. The different significance levels of these traits among different environments and genotypes were evaluated, and the statistical significance values * and ** were determined at *p* < 0.05 and *p* < 0.01, respectively. The coefficient of variation (CV) of these traits among different accessions was obtained by dividing the standard deviation by the mean value. The broad-sense heritability (*h*^2^) of these traits in different environments was calculated by the following formula:

$$h^{2}=\frac{V_{g}}{V_{g}+\frac{V_{ge}}{l}+\frac{V_{\varepsilon }}{rl}}$$

where $V_{g}$, $V_{ge}$, and $V_{\varepsilon }$ represent the genotype variance, the genotype–environment interaction variance and residual, and *l* and *r* represent the single environment repetition and environment number, respectively. Moreover, the basic correlation coefficients among these traits in two environments were calculated with Pearson approaches using the software of SPSS 19.0 (SPSS, Inc., Chicago, IL, USA), and visualization was completed by the performance analytics R package.

### 4.3. Genotyping, Population Structure, Principal Component, and Linkage Disequilibrium Analysis

The high-quality SNP variation datasets from deep resequencing were used to genotype 407 foxtail millet accessions, which were provided by Xianmin Diao, a researcher from the Institute of Crop Science, the Chinese Academy of Agricultural Sciences. After removing the low-polymorphism SNP loci with minor allele frequency (MAF) < 0.05 and missing data > 20%, high-polymorphism SNP loci were retained from 3.8 million polymorphic SNP loci for genotyping, and downstream GWAS analysis. All high-quality SNP loci were re-mapped onto the recent foxtail millet genome (*Xiaomi* Refseq) and annotated by Annovar software (v1.1) to obtain the location and relationship with gene models.

The population structure of 407 foxtail millet accessions was analyzed based on the SNP variation information using Faststructure and Structure Harvester software. The principal component analysis, phylogenetic tree, and visualization of genotype data were performed using Plink, phylip-3.697, and Evolview, respectively. The linkage relationship between the SNP loci of these genotypes was analyzed by PopLDdecay, and the linkage disequilibrium (LD) attenuation distance of thr foxtail millet was determined.

### 4.4. Genome-Wide Association Study

The high-polymorphism SNP loci filtered by the above step were used as genotypic data. The measured values of six panicle-related traits in three environments (2015, 2016, and BLUP) were used as phenotypic data and the GWAS analysis was performed using Tassel 5.0 with a mixed linear model (MLM). The Q-matrix obtained from the population structure and the Kinship matrix (K-matrix) calculated by Tassel 5.0 were used as covariates to correct the association analysis. The significance threshold level of each phenotype was determined by the replacement test (the number of replacement tests was 1000).

### 4.5. Candidate Gene Mining and Haplotype Analysis Based on Homologous Alignment

Rice (*Oryza sativa* L.) is an important model crop for the study of gene function in cereal crops. A large number of genes affecting yield and yield-related traits have been cloned in rice [21]. Following the parallel domestication of cereal crops [1], we used the strategy of rice–foxtail millet orthologous comparison to mine the genomic regions and important candidate genes affecting the panicle-related traits of foxtail millet. Through an in-depth investigation of the China Rice Research Center (https://www.ricedata.cn/ (accessed on 15 June 2022)) and previous studies, the functions of rice genes related to yield and panicle were identified and these protein sequences were extracted from the rice protein database by TBtools. The orthologous genes of foxtail millet that are homologous to rice functional genes were identified using BLASTP and MCscanX. The orthologous genes of foxtail millet, which had similar expression patterns to orthologs in rice and located in the QTL regions of the GWAS, were considered important candidate genes for regulating panicle-related traits in foxtail millet. The expression levels of genes in foxtail millet (root, stem, leaf, panicle, and developing grains from S1 to S5) and rice (root, stem, leaf, and panicle) were downloaded from the multi-omics database for *Setaria italica* (MDSi, http://foxtail-millet.biocloud.net/home (accessed on 10 May 2022)) and the rice expression database (RED, http://expression.ic4r.org/index (accessed on 10 May 2022)). The transcripts per million (TPM) values were used to assess the expression profiles of these genes, and heatmaps were drawn by log2-normalized (TPM + 1) expression levels.

## 5. Conclusions

In this study, GWAS analysis was performed for six panicle-related traits based on 706,646 high-polymorphism SNP loci in 407 foxtail millet accessions. Eighty-seven QTL regions were identified to be significantly associated with these panicle-related traits in three environments. Among these, 27 core QTL regions were detected in at least two environments. Based on homologous analysis, twenty-seven orthologous foxtail millet genes of rice genes that affected yield-related traits were identified in the QTL regions of panicle-related traits in foxtail millet. In light of this, haplotype analysis showed that 24 genes were significantly associated with panicle-related traits in foxtail millet under at least two environments. These genes regulated panicle-related traits through photoperiod response, grain development, multiple plant growth regulatory pathways, and panicle and floral organ development. In summary, this study clarified a method based on the integration of GWASs, homologous comparison, and haplotype analysis to discover the genomic regions and candidate genes for panicle-related traits in foxtail millet. This work will speed up the functional gene mining and molecular breeding efficiency of foxtail millet and other important minor cereal crops.

## Figures and Tables

**Table 1:** Descriptive statistics of six panicle-related traits of foxtail millet in two environments.

Traits	Environments	Range	Mean	SD	CV	*h^2^*	Genotype	Environment
Panicle length (PL, cm)	2015	4.50–43.90	22.20	5.50	20%	0.78	**	**
	2016	8.80–40.00	19.30	4.40	20%			
	BLUP	10.72–31.27	20.81	3.12	15%			
Main panicle diameter (MPD, mm)	2015	9.40–48.24	23.31	6.36	27%	0.74	**	**
	2016	10.19–42.82	22.63	4.69	21%			
	BLUP	14.80–35.47	22.95	3.03	13%			
Panicle weight per panicle (PWP, g)	2015	0.58–57.69	17.86	9.69	54%	0.73	**	**
	2016	1.06–35.74	14.71	5.89	40%			
	BLUP	7.64–30.45	16.26	4.32	27%			
Grain weight per panicle (GWP, g)	2015	0.50–48.88	15.06	8.51	57%	0.74	**	**
	2016	1.36–36.34	12.51	5.29	42%			
	BLUP	6.30–26.78	13.73	3.88	28%			
Bristle length (BL, mm)	2015	0.00–14.37	4.23	2.04	48%	0.91	**	*
	2016	0.00–10.48	3.86	1.76	46%			
	BLUP	0.35–10.41	4.03	1.60	40%			
Thousand-grain weight (TGW, g)	2015	0.72–3.62	2.58	0.48	19%	0.81	**	**
	2016	1.08–4.36	2.76	0.44	16%			
	BLUP	1.55–3.31	2.66	0.31	11%			

BLUP: best linear unbiased prediction; SD: standard deviation; CV: coefficient of variation; *h*^2^: broad-sense heritability; genotype and environment represent the significant levels of these traits between genotypes and environments, respectively; * and ** represent significant correlations at *p* < 0.05 and *p* < 0.01 levels, respectively.

**Table 2:** The number of significant SNP loci for six panicle-related traits by MLM model in two environments and BLUP.

Traits	Environments	Number of Significant SNPs	Range of *R^2^* (%)	Average of *R^2^* (%)
Panicle length	2015	82	0.04–12.81	4.89
	2016	111	0.25–8.04	2.62
	BLUP	371	4.06–6.63	4.90
Main panicle diameter	2015	13	0.04–20.21	4.37
	2016	41	0.06–7.89	3.45
	BLUP	55	4.03–6.67	4.72
Panicle weight per panicle	2015	231	0.44–25.85	5.69
	2016	28	0.01–12.37	6.92
	BLUP	36	4.06–5.95	4.63
Grain weight per panicle	2015	270	0.24–25.13	5.76
	2016	48	0.89–12.41	6.02
	BLUP	37	4.09–6.27	4.62
Bristle length	2015	36	0.03–26.78	16.01
	2016	24	2.33–23.30	11.91
	BLUP	320	3.99–12.44	4.91
Thousand-grain weight	2015	154	0.01–11.53	1.70
	2016	65	0.03–10.08	3.13
	BLUP	334	3.89–7.35	4.61
Total	-	1830	0.01–26.78	5.60

BLUP: the best linear unbiased prediction; *R*^2^: the phenotype interpretation rate.

**Table 3:** The core QTL regions associated with six panicle-related traits.

No.	QTL	Chr	QTL Region (Mb)	No. of QTN	QTN	Pos	*R*^2^ (%)	Trait Environment
1	PL_q2-1	2	26.405–26.442	8	2:26442425	26442425	9.30	PL.15, PL.blup
2	PL_q2-2	2	37.921–37.974	112	2:37940934	37940934	6.63	PL.16, PL.blup
3	PL_q2-3	2	38.066–38.179	37	2:38117003	38117003	4.70	PL.16, PL.blup
4	PL_q3-1	3	14.919–15.034	31	3:14994886	14994886	8.21	PL.15, PL.blup
5	PL_q3-4	3	50.531–50.635	9	3:50544489	50544489	4.80	PL.15, PL.blup
6	PL_q6-1	6	31.569–31.578	4	6:31578055	31578055	5.65	PL.16, PL.blup
7	PL_q7-1	7	13.851–13.948	26	7:13860822	13860822	12.81	PL.15, PL.blup
8	PL_q7-2	7	14.113–14.169	6	7:14120111	14120111	10.85	PL.15, PL.16, PL.blup
9	PL_q9-1	9	45.700–45.712	26	9:45712081	45712081	5.62	PL.15, PL.blup
10	PWP_q1-2	1	37.334–37.415	11	1:37334234	37334234	25.28	PWP.15, PWP.blup
11	PWP_q2-1	2	26.393–26.414	4	2:26392717	26392717	4.55	PWP.16, PWP.blup
12	PWP_q7-1	7	13.928–13.937	4	7:13927683	13927683	10.78	PWP.15, PWP.blup
13	GWP_q1-2	1	37.334–37.415	12	1:37334234	37334234	24.66	GWP.15, GWP.blup
14	GWP_q2-4	2	26.392–26.414	4	2:26392093	26392093	4.96	GWP.16, GWP.blup
15	GWP_q7-1	7	13.853–13.958	8	7:13927683	13927683	10.60	GWP.15, GWP.16, GWP.blup
16	BL_q1-4	1	30.818–30.944	16	1:30890043	30890043	12.67	BL.15, BL.16, BL.blup
17	BL_q1-5	1	31.336–31.388	19	1:31384418	31384418	13.65	BL.15, BL.blup
18	BL_q1-6	1	37.312–37.386	190	1:37322139	37322139	23.46	BL.15, BL.16, BL.blup
19	BL_q1-7	1	37.626–37.728	39	1:37724771	37724771	24.65	BL.15, BL.16, BL.blup
20	BL_q1-8	1	37.730–37.818	62	1:37751602	37751602	24.16	BL.15, BL.16, BL.blup
21	BL_q1-9	1	37.854–37.942	15	1:37855555	37855555	12.44	BL.15, BL.16, BL.blup
22	BL_q1-10	1	38.004–38.100	4	1:38099726	38099726	23.57	BL.15, BL.blup
23	BL_q1-11	1	38.240–38.293	20	1:38251263	38251263	21.12	BL.15, BL.16, BL.blup
24	BL_q4-1	4	0.833–0.893	6	4:893187	893187	7.54	BL.16, BL.blup
25	BL_q8-1	8	1.635–1.668	18	8:1635250	1635250	8.93	BL.16, BL.blup
26	BL_q9-1	9	42.909–42.944	20	9:42943571	42943571	14.14	BL.15, BL.blup
27	TGW_q5-1	5	0.546–0.549	4	5:545898	545898	8.32	TGW.16, TGW.blup

Chr: chromosome; QTN: quantitative trait nucleotide; Pos: position; *R*^2^ (%): the highest phenotype interpretation rate in QTL region; Trait_environment: the trait–environment combination of QTN.

**Table 4:** The overlapped QTLs between present and previous studies.

QTL Regions	Chr	QTL Region (Mb)	Previous Reported QTL	Position of Previous QTL (Mb)	Ref.
BL_q1-5	1	31.336–31.388	qMPL1.1 (PL), qMPD1.1 (PD), qPWP1.1 (PWP), qGWP1.1 (GWP), qpl1 (PL), qpd1 (PD), C1.31690904 (GY)	31.728–31.929	[15,23,25]
PWP_q1-1, GWP_q1-1	1	34.970–34.987	unnamed (BL)	35.883–35.869	[24]
PL_q2-2, PL_2-3	2	37.921–38.179	qMPL2.1 (PL)	39.726	[15]
PL_q2-4	2	49.117–49.200	unnamed (GY)	49.065	[24]
TGW_q3-1	3	6.258–6.342	unnamed (PWP)	6.768	[24]
PWP_q3-1, GWP_q3-1	3	7.353–7.409	qGWP3.1 (GWP), unnamed (TGW)	7.412–7.806	[24,26]
PL_q3-1	3	14.919–15.034	unnamed (PL)	14.391	[23]
MPD_q3-1, TGW_q3-2	3	23.616–23.733	unnamed (GY)	24.672	[24]
PL_q3-2	3	39.875–39.995	qPWP3.1 (PWP), qGWP3.2 (GWP)	39.037	[26]
TGW_q3-3	3	43.160–43.182	unnamed (PL), unnamed (TGW)	43.132–43.267	[23,24]
MPD_q3-2	3	47.020–47.023	qPWP3.3 (PWP)	46.338–46.477	[26]
PL_q3-4	3	50.531–50.635	unnamed (GY)	50.073	[23]
BL_q4-1	4	0.833–0.893	unnamed (BL)	0.871	[24]
MPD_q4-1	4	34.740–34.746	qtgw4 (TGW)	34.306	[25]
MPD_q5-2	5	45.138–45.166	qMPD5.2 (MPD), qTGW5.1 (TGW), qpw5 (PWP), qpd5(PD)	44.125–44.511	[15,25]
PWP_q6-1, GWP_q6-1, PWP_q6-2, GWP_q6-2	6	2.535–2.794	qPWP6.2 (PWP), unnamed (GY)	2.631–3.434	[23,26]
PWP_q6-7, GWP_q6-7, GWP_q6-9, PWP_q6-8, GWP_q6-9	6	27.924–28.247	qPNL6 (PL)	28.589	[16]
TGW_q6-1	6	34.670–34.733	qMPD6.1 (PD)	34.199	[15]
PL_q7-1, TGW_q7-1, PWP_q7-1, GWP_q7-1	7	13.851–13.961	qGWP7.1 (GWP), qPWP7.2 (PWP)	13.405–13.643	[26]
PL_q7-2, TGW_q7-2, TGW_q7-3	7	14.113–14.893	unnamed (GY)	14.094	[23]
TGW_q7-8	7	18.967–19.040	unnamed (GY)	19.465	[23]
GWP_q8-1	8	2.181–2.191	qGWP8.1 (GWP), qTGW8.1 (TGW)	2.223–2.605	[26]
TGW_q8-1	8	28.829–28.851	qPD8.1 (PD)	29.501	[16]

Chr: chromosome; Ref: reference; PL, MPD, PWP, GWP, BL, GY, and TGW represent the panicle length, main panicle diameter, panicle weight per panicle, grain weight per panicle, bristle length, grain yield, and thousand-grain weight, respectively.

**Table 5:** The details of 27 candidate genes associated with panicle-related traits in foxtail millet.

Gene ID	Chr	Position	QTL Region	Homologs in Rice	The Function of Orthologue in Rice
Si1g24310	1	31.649-31.652	BL_q1-5	OsPRR1 (LOC_Os02g40510) [27]	Internode length, spike length, tiller number, grain size, and number of primary branches
Si1g27910	1	34.867-34.874	GWP_q1-1 PWP_q1-1	OsMADS6 (LOC_Os02g45770) [28]	Protein content, sterility or low fertility, gelatinization temperature, amylose content, seed setting rate, grain width, grain length, floral organ development characteristics, and total starch content
Si1g31910	1	37.921-37.926	BL_q1-10	OsGRF4; GS2; GL2; PT2; LGS1; GLW2(LOC_Os02g47280) [29]	Grain shape, ear length, grain size, grain yield, seed drop, cold tolerance, grain width, grain length, plant dry weight, 1000-grain weight, plant cell size, plant fresh weight, and nitrogen use efficiency
Si1g34880	1	40.251-40.252	PL_q1-2	OsMKK4; SMG1 (LOC_Os02g54600) [30]	Plant height, grain size, panicle type, filled grains per panicle, grain width, grain length, lignin content, cytokinin content, diterpenoid phytoalexin content, and 1000-grain weight
Si2g10220	2	9.256-9.262	GWP_q2-3	LLB; pls3; OsMTS1 (LOC_Os07g14350) [31]	Rice blast resistance, bacterial blight resistance, leaf color, chlorophyll content, grain yield, leaf inclination, leaf senescence, lesion-like lesion, hydrogen peroxide, jasmonic acid, and aboveground biomass
Si2g27120	2	36.896-36.902	BL_q2-1	qGW8; OsSPL16 (LOC_Os08g41940) [32]	Grain shape, cooking quality, grain size, mitotic cycle, and 1000-grain weight
Si2g43940	2	49.297-49.308	PL_q2-4	DTH7; Ghd7.1; OsPRR37 (LOC_Os07g49460) [33]	Plant height, heading date, photoperiod sensitivity, growth period, and grain number per panicle
Si3g11190	3	7.375-7.379	PWP_q3-1 GWP_q3-1	GSE5; GW5 (LOC_Os05g09520) [34]	Grain shape, grain size, grain width, grain length, and 1000-grain weight
Si3g11200	3	7.390-7.394	PWP_q3-1 GWP_q3-1	OsDER1 (LOC_Os05g09550) [35]	Seed development characteristics, grain width, grain length, and 1000-grain weight
Si3g33230	3	43.050-43.055	TGW_q3-3	OsCSLD4; NRL1; ND1; OsCD1 (LOC_Os12g36890) [36]	Plant height, leaf width, ear length, leaf inclination, leaf roll, grain number per ear, and sugar content
Si4g02810	4	1.359-1.363	TGW_q4-1	OsACS6; SSG6 (LOC_Os06g03990) [37]	Grain size, grain width, grain length, starch granule shape, 1000-grain weight, chloroplast development, and starch granule size
Si4g23080	4	34.377-34.386	MPD_q4-1	OsARF19; OsARF7a (LOC_Os06g48950) [38]	Internode length, leaf width, leaf length, grain yield, leaf inclination, floral organ development characteristics, auxin content, 1000-grain weight, and plant cell size
Si5g01910	5	0.793-0.800	TGW_q5-1	DFO1; CCP1 (LOC_Os01g12890) [39]	Plant height, male sterility, seed morphology and anatomy, ear length, tiller number, lemma morphology and anatomy, seed setting rate, and primary branch number
Si5g07490	5	5.957-5.958	PL_q5-1	OsLIR1 (LOC_Os01g01340) [40]	Vegetative growth potential, seed setting rate, photosynthetic capacity, and spikelet fertility
Si5g07510	5	5.969-5.971	PL_q5-1	OsNPF6.1 (LOC_Os01g01360) [41]	Plant height, panicle number, nitrogen content, nitrate transport, yield per plant, and nitrogen use efficiency
Si5g10260	5	8.652-8.657	PWP_q5-1	OsSQD2.2 (LOC_Os01g04920) [42]	Tiller number, seed setting rate, total starch content, and total soluble sugar content
Si6g16030	6	27.100-27.101	PWP_q6-3 GWP_q6-3	ONAC106 (LOC_Os08g33670) [43]	Leaf color, fertility, ear length, grain yield, salt tolerance, tiller angle, ear number, grain number per ear, oriented gravity, and 1000-grain weight
Si6g20480	6	31.677-31.680	PL_q6-1	OsSPL14; IPA1; WFP (LOC_Os08g39890) [44]	Rice blast resistance, bacterial blight resistance, plant height, ear length, tiller number, grain yield, ear branches, grain number per ear, seed setting rate, lodging rate, stem diameter, and 1000-grain weight
Si6g23990	6	34.326-34.327	TGW_q6-1	OsSAUR45 (LOC_Os09g37400) [45]	Adventitious root number, plant height, root length, leaf width, and seed setting rate
Si7g07780	7	15.603-15.606	TGW_q7-7	An-1 (LOC_Os04g28280) [46]	Awn length, grain number per ear, and grain length
Si7g10650	7	19.276-19.279	TGW_q7-8	GIF1; OsCIN2 (LOC_Os04g33740) [47]	Viscous endosperm, grain size, grain yield, amylose content, amylopectin content, and invertase activity
Si7g20960	7	27.099-27.101	MPD_q7-1	OsPIP1.1; OsPIP1a (LOC_Os02g44630) [48]	Grain yield, salt tolerance, germination rate, and germination rate
Si7g27560	7	32.242-32.246	PL_q7-4	GS5 (LOC_Os05g06660) [49]	Grain size, characteristics of seed development, grain yield, grain width, and 1000-grain weight
Si8g15190	8	28.271-28.275	TGW_q8-1	OsFKF1 (LOC_Os11g34460) [50]	Fertility, heading date, number of panicles per plant, number of grains per panicle, and 1000-grain weight
Si9g12860	9	8.332-8.335	PWP_q9-1	OsNF-YA4; OsHAP2D (LOC_Os03g48970) [51]	Number of ears, number of ears, number of grains per ear, and grain weight
Si9g13550	9	8.916-8.920	GWP_q9-1	OsFdC2 (LOC_Os03g48040) [52]	Plant height, leaf color, heading date, ear number per plant, grain number per ear, seed setting rate, photosynthetic capacity, carotenoid content, chlorophyll a content, chlorophyll b content, chlorophyll a/b ratio, and chloroplast development
Si9g39120	9	45.984-45.994	PL_q9-1	VLN2 (LOC_Os03g24220) [53]	Morphological and anatomical characters of root, plant height, leaf angle, panicle type, seed setting rate, grain width, grain thickness, and 1000-grain weight

Chr: chromosome.

## Data Availability

Not applicable.

## Supplementary Materials

The following supporting information can be downloaded at: https://www.mdpi.com/article/10.3390/ijms232314735/s1.
